# Polysaccharide-based Noncovalent Assembly for Targeted Delivery of Taxol

**DOI:** 10.1038/srep19212

**Published:** 2016-01-13

**Authors:** Yang Yang, Ying-Ming Zhang, Yong Chen, Jia-Tong Chen, Yu Liu

**Affiliations:** 1 Department of Chemistry, State Key Laboratory of Elemento-Organic Chemistry, Nankai University, Tianjin, 300071, P. R. China; 2 Collaborative Innovation Center of Chemical Science and Engineering (Tianjin), Nankai University, Tianjin, 300071, P. R. China; 3 School of Chemical Engineering and Technology, Hebei University of Technology, Tianjin, 300130, P. R. China; 4 Department of Biochemistry and Molecular Biology, College of Life Sciences, Nankai University, Tianjin, 300071, P. R. China

## Abstract

The construction of synthetic straightforward, biocompatible and biodegradable targeted drug delivery system with fluorescent tracking abilities, high anticancer activities and low side effects is still a challenge in the field of biochemistry and material chemistry. In this work, we constructed targeted paclitaxel (Taxol) delivery nanoparticles composed of permethyl-*β*-cyclodextrin modified hyaluronic acid (HApCD) and porphyrin modified paclitaxel prodrug (PorTaxol), through host-guest and amphiphilic interactions. The obtained nanoparticles (HATXP) were biocompatible and enzymatic biodegradable due to their hydrophilic hyaluronic acid (HA) shell and hydrophobic Taxol core, and exhibited specific targeting internalization into cancer cells via HA receptor mediated endocytosis effects. The cytotoxicity experiments showed that the HATXP exhibited similar anticancer activities to, but much lower side effects than commercial anticancer drug Taxol. The present work would provide a platform for targeted paclitaxel drug delivery and a general protocol for the design of advanced multifunctional nanoscale biomaterials for targeted drug/gene delivery.

The construction of novel diagnosis and drug-therapy systems against cancer nowadays becomes research hotspots in the fields of biochemistry and material chemistry^1^,^2^,^3^. For this purpose, a number of targeted drug delivery systems based on inorganic nanoparticles^4^,^5^,^6^, carbon nano-materials^7^,^8^,^9^, liposomes^10^,^11^,^12^, and vesicles^13^,^14^,^15^ were constructed and exhibited significant therapeutic activities toward cancer cells and tissues *in vitro* and *in vivo*. However, the complicated synthetic routes, multi-stepped modification and separation, insufficient targeting capability, undefined mechanism of drug release and cytotoxicity of the drug carriers would not facilitate their benefits in anticancer therapy and therefore impede their practical applications in clinic.

On the other hand, the early diagnosis of cancer plays an important role in prevention, early detection and early treatment of cancer and the early find of cancer might promote the survival rate of the patients suffered from cancer. Therefore, the development of novel and sensitive diagnostic reagents toward cancer also draw attention of the interest of medical researchers. Therefore a large amount of fluorescent^16^,^17^,^18^,^19^, photoacoustic^20^,^21^,^22^, and magnetic imaging reagents^23^,^24^,^25^ were synthesized and then revealed excellent and sensitive detective viability to cancer cells and tissues specifically. However, the construction of multi-functional integrated diagnosis/imaging and targeted drug delivery systems with multifarious therapeutic approaches and controllable drug release through relative simple synthetic methods is still scarce and imperative, and this kind of delivery systems is anticipated to be applied in clinic practically.

Importantly, the convenient construction and controllable drug release of targeted drug delivery system could be probably realized by supramolecular chemistry and amphiphilic interactions without complicated modification process^26^,^27^,^28^. The ultra-strong host-guest interaction between permethyl-*β*-cyclodextrin (permethyl-*β*-CD) and porphyrin derivates had been reported in numerous supramolecular architectures^29^,^30^,^31^, and the changes of pH value and polarity of the solution might induce the disassembly of the architectures. Moreover, hyaluronic acid (HA) was used as carrier and targeting reagent in targeted drug delivery systems due to its biocompatibility, biodegradability, easy to modification, and specific binding with CD44 and RHAMM receptors over expressed on the surface of cancer cells^32^,^33^. Very recently, quantities of research groups took advantage of modified HA as carriers and targeting reagents to realize the satisfactory drug delivery efficiency into cancer cells and tissues *in vitro* and *in vivo* without obvious side effects. For instance, Chen *et al.*^34^ developed a robust tumor-targeted and pH-responsive nanoplatform containing 5*β*-cholanic acid/Zn^II^-dipicolylamine tethered HA and pH-dependent CaP shell, and the obtained nanoparticle could strongly bind anticancer drug doxorubicin and multidrug resistance 1 gene target siRNA, and then after associating with the CD44 receptor over-expressed cancer cells, the effective drug/siRNA release and the targeted cytotoxicity toward drug resistant cancer cells could be realized *in vitro* and *in vivo* due to the suppression of target gene expression. Furthermore, Hahn *et al.*^35^ covalently connected HA molecules onto the surface of graphene oxide, then the biostable and biocompatible graphene oxide with tumor-targeting function could enter into melanoma skin cancer cells selectively and the photothermal ablation of tumor could be achieved under the irradiation of near infrared laser light. Kim *et al.*^36^ reported a targeted nanoparticles by poly-cyclodextrin and poly-paclitaxel interactions, and the nanoparticle was enzyme-degradable and exhibited targeted toxicity toward cancer cells and tissues *in vitro* and *in vivo*. However, the relative complicated synthetic routes would not facilitate the practical application of the nanoparticles in clinic. Previously our group^37^ have constructed a safe and simple nanoparticle containing *β*-cyclodextrin modified HA and adamantane modified cisplatin prodrug via host-guest and amphiphilic interactions, and the obtained nanoparticle could specifically target HA-receptor positive cancer cells, and exhibit better anticancer activities and lower side effects than commercial chemotherapy drug cisplatin *in vitro* and *in vivo*.

In this work, we synthesized permethyl-*β*-CD modified HA (HApCD) and porphyrin modified paclitaxel (PorTaxol) through amide condensation reaction and esterification reaction, respectively. Taking advantage of the ultra-strong host-guest interaction between permethyl-*β*-CD and porphyrin part, we got a supramolecular complex with amphiphilicity, and this amphiphilic complex could self-assemble to form nanoparticles HATXP which had a hydrophilic HA shell that could target HA-receptor over-expressed on the surface of cancer cells, and a hydrophobic PorTaxol prodrug core that possessed fluorescent labeled function (Fig. 1). The HATXP nanoparticles were stable, biocompatible, biodegradable, and nano-sized, and exhibited similar anticancer activities to, but much lower side effects than commercial anticancer drug paclitaxel (Taxol).

## Results and Discussion

### Synthesis

The synthetic routes of HApCD and the porphyrin modified paclitaxel (PorTaxol) were shown in Supplementary Fig. S1-S2. HApCD was synthesized by the amide condensation reaction of hyaluronic acid sodium with mono-6-deoxyl-6-ethylenediamino-permethyl-*β*-CD in phosphate buffer solution (PBS) and characterized by ^1^H NMR spectroscopy and gel permeation chromatography (GPC). As shown in Supplementary Fig. S3-S4, 1H NMR spectra of HA and HApCD showed an obvious characteristic signal assigned to H1 protons of permethyl-*β*-CD appeared around the chemical shift of 5 ppm, and the signals assigned to H2-H6 protons of permethyl-*β*-CD’s and HA’s protons appeared around 3–4 ppm, which both indicated the successful grafting of permethyl-*β*-CD onto the skeleton of HA molecule. After analyzing the integral values of the peak area of permethyl-*β*-CD’s and HA’s protons, we could calculate that there is one permethyl-*β*-CD modified on every 7.37 polysaccharide units of HA, and this degree of substitution (DS) may not impede the targeting properties of HA backbone toward CD44 receptors overexpressing on the surface of malignant cancer cells which require at least six repeating units to form a targeting section^38^. Under this DS value, we can calculate the relative molecular weight of HApCD as 2.72 × 10^5^, which is very consistent with the weight-average molecular weight (*M*_w_, which was determined by SEC columns with reference to poly(ethylene oxide), see Supplementary Fig. S5-S6) as 2.83 × 10^5^ determined by GPC. The increasing molecular weight of HApCD compared with HA (*M*_w_ = 1.9 × 10^5^) further confirms the successful modification of permethyl-*β*-CD onto HA molecule. The characterization data of HApCD from ^1^H NMR and GPC were summarized in Table 1. On the other hand, PorTaxol was synthesized by esterification reaction of Taxol with 5-(4-carboxyphenyl)-10,15,20-triphenylporphyrin in 45% yield. As shown in Supplementary Fig. S7-S8, the chemical shift of hydrogen on C-*α* in Taxol showed a large shift from 4.96 ppm to 5.74 ppm after the modification of Taxol with porphyrin, which revealed that the carboxyl group of porphyrin molecule reacted with the hydroxide group of Taxol on C-*α*, and then lead to the changes in the chemical environment of H-*α*.

### Complexation of PorTaxol with HApCD

**Figure 2:**
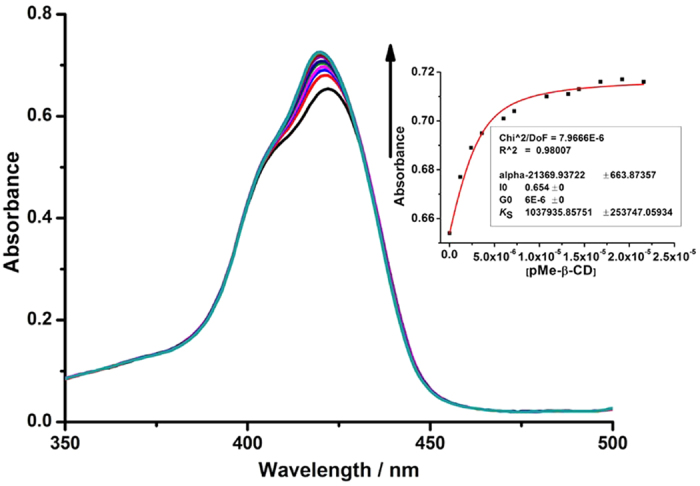
UV/Vis titration of PorTaxol prodrug by permethyl-*β*-CD. UV/Vis spectra of PorTaxol (6.0 × 10^−6^ M) upon addition of permethyl-*β*-CD (0–2.16 × 10^−5^ M) in PBS (pH = 7.2, containing 5% DMSO). Insert: nonlinear least-squares fit of the absorbance changes (*λ* = 422 nm) of PorTaxol as a function of the permethyl-*β*-CD concentration.

The UV/Vis spectral titration was carried out to investigate the association between PorTaxol and the permethyl-*β*-CD cavity in HApCD. As shown in Fig. 2, the Soret band of PorTaxol showed a hypsochromic shift from 422 nm to 420 nm with the addition of permethyl-*β*-CD, indicating that the porphyrin backbone in PorTaxol was included into the permethyl-*β*-CD cavity^39^. Meanwhile, the Job’ plot demonstrated a 1:1 binding stoichiometry between PorTaxol and permethyl-*β*-CD cavity (Supplementary Fig. S11). Therefore, the association constant (*K*_S_) between PorTaxol and permethyl-*β*-CD was calculated as (1.04 ± 0.25) × 10^6^ M^−1^, according to the results in the UV/Vis spectral titration by analyzing the sequential changes of the absorption intensity of PorTaxol at varying the concentrations of permethyl-*β*-CD.

### Characterization of HATXP

Taking advantage of the strong affinity between porphyrin unit in PorTaxol and the permethyl-*β*-CD cavity in HApCD, a supramolecular nanoparticle (HATXP) containing hyaluronic acid as a targeting unit and Taxols as bioactive units was successfully constructed by simply mixing the HApCD and PorTaxol in aqueous solution. Then the obtained mixture was dialyzed against an excess amount of deionized water for 2 h to remove any unbounded PorTaxol. Then the drug loading ratio (the weight ratio of loaded drug to carrier) and drug encapsulation ratio of PorTaxol were measured as 31% and 85%, respectively, through the photometric standard curve of PorTaxol at *λ* = 515 nm (Supplementary Fig. S12). The high drug loading ratio and drug encapsulation ratio were attributed to the strong interactions between porphyrin and permethyl-*β*-CD as well as the strong amphiphilic interactions in HATXP, which could prevent the PorTaxol running out of the HATXP particles.

**Figure 3:**
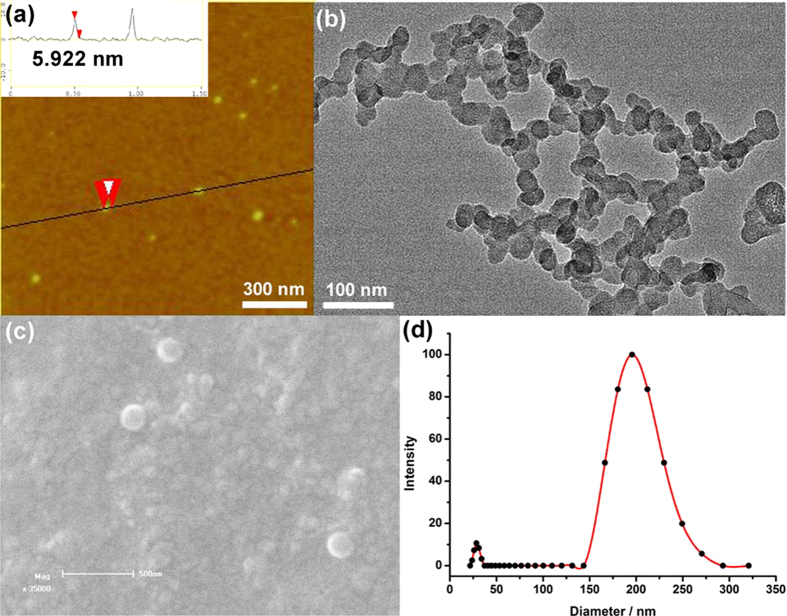
Microscope and DLS characterization of HATXP. Typical (**a**) AFM, (**b**) HR-TEM, and (**c**) SEM images of HATXP, and (**d**) DLS result of HATXP in PBS.

Then the size and morphology of HATXP were measured by atomic force microscopy (AFM), high-resolution transmission electron microscopy (HR-TEM), scanning electron microscopy (SEM), dynamic light scattering (DLS), and zeta potential experiments. As shown in Fig. 3a, a number of discrete spherical nanoparticles whose diameters distributed from 30 to 40 nm were observed in a typical AFM images, and the height of the collapsed nanoparticle was measured as *ca.* 5.9 nm, which was closely equal to the sum of two HA backbones (1.8 nm), two permethyl-*β*-CDs (2.0 nm), and two Taxol molecules (2.0 nm)^37^. HR-TEM image (Fig. 3b) also showed the morphology of HATXP as spherical nanoparticles with an average diameter of 30 ~ 50 nm, which is basically consistent with the results obtained in AFM image. In addition, the HATXP nanoparticles also showed a tendency of self-aggregating to larger assemblies through the inter-particle hydrogen-bonding interactions among numerous carboxyl and hydroxyl groups on the backbone of HA. This tendency of self-aggregate was also supported by SEM image (Fig. 3c) that presented many aggregated spherical nanoparticles. The control experiment showed that, without PorTaxol, the free HApCD only existed as amorphous structures in AFM, TEM, and SEM (Supplementary Fig. S13). Moreover, DLS experiments gave a hydrodynamic diameter of HATXP as *ca.* 171 nm with a narrow distribution (Fig. 3d). The zeta potential of HATXP was measured as *ca.* −29 mV (Supplementary Fig. S14), which was similar to the corresponding value of HApCD (*ca.* −34 mV) or HA (−15 mV) (Supplementary Fig. S15–16) owing to the ionization of carboxyl groups on the HA skeleton, and the negative charge on the surface of HATXP would facilitate the stability, dispersibility, and biocompatibility of HATXP in biological environment and prolong the circulation time of HATXP *in vivo*^37^, eventually availing the delivery and release of PorTaxol. In addition, the formation of HATXP nanoparticles could also be distinguished through Tyndall effects by naked eyes. As shown in Supplementary Fig. S17, the aqueous solution of HApCD exhibited the very weak Tyndall effect, but presented the strong Tyndall effect after the addition of PorTaxol.

The biocompatibility of PorTaxol and HATXP were measured comparatively in PBS. As shown in Supplementary Fig. S18, PorTaxol could dispersed well in water (5% DMSO), but precipitated immediately in PBS (5% DMSO). After the addition of HApCD, the negative charged HA shell of HATXP would protect PorTaxol from the coagulation and then facilitate the dispersion of PorTaxol in PBS, and thus a clear and transparent solution could be obtained. A possible reason may be that, owing to the strong association of permethyl-*β*-CD with porphyrin, HApCD and PorTaxol could form an amphiphilic complex, which further assembled to nanoparticles possessing the hydrophobic PorTaxol core and the hydrophilic HA shell with great biocompatibility and targeting capability toward cancer cells.

**Figure 4:**
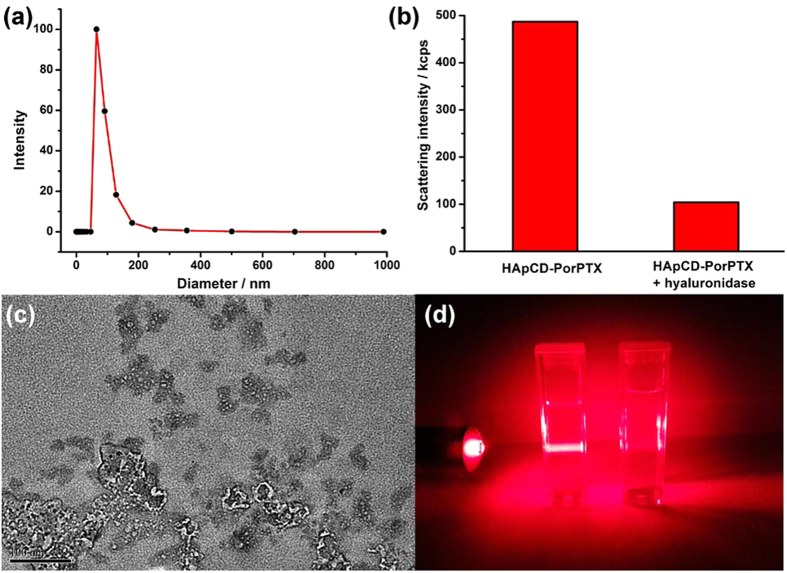
Enzymatic hydrolysis of HATXP by hyaluronidase. (**a**) DLS result of hydrodynamic diameter and (**c**) TEM image of HATXP after hydrolyzation by hyaluronidase for 2 h. (**b**) Scattering-light intensity and (**d**) Tyndall effects of HATXP before (left) and after (right) degradation by hyaluronidase for 2 h.

Moreover, the release of PorTaxol caused by hyaluronidase-mediated hydrolyzation was also investigated. As shown in Fig. 4a, after treated with hyaluronidase at 37 °C in PBS, HATXP still gave a hydrodynamic diameter as 176 nm in DLS experiments, but the scattering-light intensity greatly decreased by *ca.* 80%, indicating that a majority of HATXP was biodegraded by hyaluronidase (Fig. 4b). Moreover, no spherical nanoparticle assigned to HATXP, but only the amorphous structure assigned to the free PorTaxol, could be observed in HR-TEM (Fig. 4c). These phenomena, along with the disappearance of Tyndall effect (Fig. 4d), jointly demonstrated that the HA shell of HATXP could be hydrolyzed to low-molecular-weight oligomers through the breaking of endo-*N*-acetylhexosaminic bonds in HA chains upon interaction with hyaluronidase, leading to the biodegradation of HATXP^40^.

### Fluorescence confocal imaging

**Figure 5:**
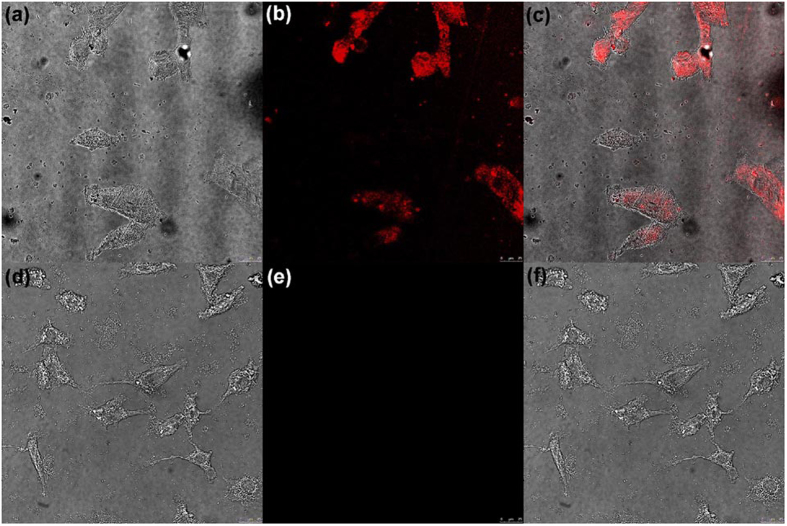
Fluorescent confocal experiments. Fluorescent confocal images of SKOV-3 cells in (**a**) bright field, (**b**) dark field, (**c**) merged field, and NIH3T3 cells in (**d**) bright field, (**e**) dark field, (**f**) merged field after incubation with HATXP for 6 h.

Furthermore, the HA-mediated cell uptake of HATXP was also investigated by means of fluorescent confocal image experiments using SKOV-3 human ovarian cancer cell line (HA-receptor positive) and NIH3T3 mouse embryonic fibroblast cell line (HA-receptor negative) as model cells. As shown in Fig. 5, after incubation with HATXP for 6 h, SKOV-3 cancer cells exhibited typical red fluorescence of porphyrin^30^. Lacking the HA-receptor on the cell membrane, NIH3T3 cells exhibited very weak fluorescence of porphyrin. Moreover, the cytotoxicity experiments discussed below showed that the PorTaxol could both enter into SKOV-3 and NIH3T3 cells, and then induced the cytotoxic effects. These phenomena jointly indicated that the HATXP nanoparticles could be effectively and selectively ingested by the HA-receptor-positive cancer cells, but not by the HA-receptor-negative normal cells, through the HA-receptor mediated cellular endocytosis. Then, in the intracellular environment where hyaluronidase was over-expressed^41^,^42^, HATXP could be disassembled through the enzymatic hydrolysis to PorTaxol. Therefore, HATXP can not only be used as the diagnosis and tracer reagent for cancer cells and tissues, but also a therapeutic reagent to suppress the proliferation of cancer cells with high efficiency and without cytotoxicity to normal cells, which would be described below.

### Cytotoxicity experiment

**Figure 6:**
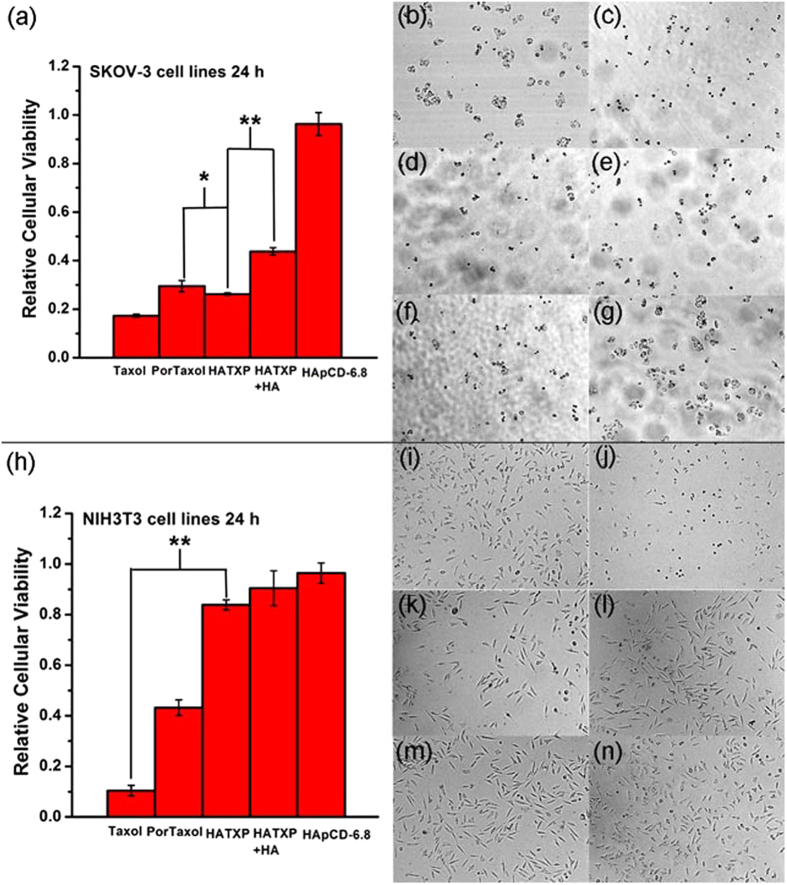
Cytotoxicity experiments *in vitro* in 24 h. Cytotoxicity experiment results of (**a**) SKOV-3 cells and (**h**) NIH3T3 cells in 24 h, SKOV-3 cell images of (**b**) blank control; (**c**) Taxol; (**d**) PorTaxol; (**e**) HATXP; (**f**) HATXP+HA; (**g**) HApCD, and NIH3T3 cell images of (**i**) blank control; (**j**) Taxol; (**k**) PorTaxol; (**l**) HATXP; (**m**) HATXP+HA; (**n**) HApCD. The differences between PorTaxol and HATXP, HATXP and HATXP + HA for SKOV-3 cells, and Taxol with HATXP for NIH3T3 cells that were statistically significant are indicated with asterisk (P < 0.05) and double asterisks (P < 0.005).

The anticancer effect of HATXP nanoparticles was evaluated by the cytotoxicity experiments. As shown in Fig. 6a, the commercial anticancer drug Taxol exhibited the satisfactory cytotoxicity towards SKOV-3 cells with a relative cellular viability of 17%, and PorTaxol gave a slightly lower anticancer activity with a relative cellular viability of 29% towards SKOV-3 cells. Possessing a hydrophobic anticancer drug core and a hydrophilic HA shell with targeting effects to cancer cells, HATXP could enter the SKOV-3 cancer cells through the HA-mediated endocytosis and then release the ingested drug through the interactions of hyaluronidase and esterase. This targeting effect could increase the drug concentration in cancer cells and ultimately promote the anticancer effect. As a result, HATXP gave a relative cellular viability as 26% towards SKOV-3 cells, which was little better than that of PorTaxol. However, the anticancer activity of HATXP greatly decreased (the relative cellular viability was 44% towards SKOV-3 cells) when SKOV-3 cells were treated with an excess amount of HA. A possible reason may be that the saturation of HA-receptor on the surface of cancer cells with free HA molecules disfavored the interaction of HA-receptors with HA shell of HATXP, which further certificated that the interaction of HA shell of HATXP with the HA-receptors on the surface of cancer cells played an important role in the internalization process of HATXP into cancer cells. Moreover, the similar anticancer activities between Taxol and HATXP could also be verified by IC50 values (half maximal inhibitory concentration). As shown in Supplementary Fig. S19, the IC50 values for Taxol alone and PorTaxol in HATXP particles in 24 h were measured as 2.91 *μ*M and 2.89 *μ*M, respectively, which would further prove our conclusion that HATXP showed comparable anticancer activity to commercial Taxol.

It is noteworthy that, although HATXP showed a bit lower anticancer activity than Taxol (relative cellular viability 26% by HATXP vs 17% by Taxol towards SKOV-3 cells), its toxicity towards normal cells (relative cellular viability 84% by HATXP vs 10% by Taxol towards NIH3T3 cells) was very lower than that of Taxol (Fig. 6h). The results of cytotoxicity experiments in 48 h (Supplementary Fig. S20) were basically consistent with the ones in 24 h. Control experiments also showed that HApCD displayed no obvious toxicity to either SKOV-3 cancer cells or NIH3T3 normal cells. Moreover, the morphological changes of SKOV-3 cells (Fig. 6b–g) and NIH3T3 cells (Fig. 6i–n) treated with different reagents showed that, towards cancer cells, HATXP presented the similar damaging effect to Taxol but was less toxic toward normal cells than Taxol.

## Conclusion

In conclusion, a targeted nanoparticle with hydrophobic PorTaxol core and hydrophilic HA shell was constructed through supramolecular and amphiphilic interactions. The nanoparticles could specifically recognize malignant cancer cells through HA-HA receptor interactions and induce the apoptosis of cancer cells without obvious side effects toward normal cells. On the other hand, taking advantage of the fluorescent porphyrin part, the location of the nanoparticles in cells could be explicit. Therefore, these nanoparticles could act as an integrative diagnosis-treatment platform, and the potential applications of nanoparticles in photodynamic therapy are still in process.

## Methods

### Materials

All chemicals were reagent-grade unless noted otherwise. *β*-CD was recrystallized twice from water and dried *in vacuo* at 90 °C for 24 h prior to use. Sodium hyaluronate (M_w_ = 190,000), paclitaxel (Taxol), 1-ethyl-3-(3-dimethylaminopropyl)-carbodiimide (EDC), *N*-hydroxysulfosuccinimide (NHSS), *N,N*′-dicyclohexyl-carbodiimide (DCC), 4-dimethylaminopyridine (DMAP), and hyaluronidase were purchased from commercial sources and used as received. Mono-6-deoxyl-6-ethylenediamino-permethyl- *β*-CD^43^, and 5-(4-carboxyphenyl)-10,15,20-triphenylporphyrin^44^ were prepared according to procedures reported. Crude chloroform was stirred with CaH_2_ for 1 day and then distilled at room pressure prior to use. Column chromatography was performed on 200–300 mesh silica gel.

### Instrument

NMR spectra were recorded on a Bruker AV400 instrument. UV/Vis spectra were recorded in a conventional quartz cell (light path 10 mm) by using a Shimadzu UV-2401PC spectrophotometer equipped with a Thermo HAAKE-SC100 temperature controller to keep the temperature at 25 °C. For the AFM measurements, a sample solution (0.1 mg/mL) was dropped onto newly clipped mica and air-dried, and the residue obtained was examined in tapping mode in the air under ambient conditions using a Veeco Nano IIIa Multimode AFM instrument. High-resolution transmission electron microscope (HR-TEM) images were obtained on a Tecnai G^2^ F20 microscope instrument operated at 200 kV. The samples were prepared by placing a drop of solution (0.1 mg/mL) onto a carbon-coated copper grid. The samples for SEM measurements were prepared by dropping each sample solution onto a coverslip followed by evaporation of the solvent at room temperature. SEM images were obtained on a Shimadzu SS-550 scanning electron microscope. The sample solutions for DLS experiments were prepared by filtering each solution through a 450 nm syringe-driven filter (JET BIOFIL) into a clean scintillation vial. The samples were examined on a NanoBrook 173Plus at *λ* = 636 nm at 25 °C. All DLS measurements were performed at the scattering angle of 90°. The zeta potential was recorded on a NanoBrook 173Plus at 25 °C. GPC measurements were employed to examine the weight-averaged molecular weights (*M*_*w*_) of polymers on a four detection size exclusion chromatograph (Four-SEC) containing a Waters 1525 separation module connected with a Viscotek M302 four detector array, a combination of refractive index, light scattering (LS angle, 7 and 90°, laser wavelength, *λ* = 532 nm), viscosity detector, and UV/Vis detector. Two mixed bed SEC columns (GMH_HR_-M, GMH_HR_-H from Viscotek) were used. Poly(ethylene oxide) was used as calibration standard and 0.1 M phosphate buffer (pH = 7.2) was used as a mobile phase at a flow rate of 1.0 mL min^−1^ at an operating temperature of 25 °C. The fluorescence confocal images were carried out on Leica TCS SP8 fluorescence microscope (*λ*_ex_ = 458 nm, 25 °C).

### Synthesis of permethyl-*β*-CD modified hyaluronate acid (HApCD)

1-Ethyl-3-(3-dimethyl aminopropyl)-carbodiimide (EDC) (167.7 mg, 0.875 mmol) and *N*-hydroxysulfosuccinimide sodium salt (NHSS) (190 mg, 0.875 mmol) were added to a solution of sodium hyaluronate (*M*_*w*_ = 190,000, 100 mg, 0.53 *μ*mol) in phosphate buffer saline (PBS, 0.1 M, pH = 7.2, 30 mL) and the mixture was stirred at 25 °C for 30 min, then mono-6-deoxyl-6-ethylenediamino- permethyl-*β*-CD (364.4 mg, 0.25 mmol) in PBS (10 mL) was added. The reaction mixture was stirred at room temperature for 24 h. The resulting solution was dialyzed against excess amount of deionized water for 5 days. After being freeze-dried, the product HApCD was obtained as a white powder. ^1^H NMR (400 MHz, D_2_O, TMS): *δ* = 1.87 (s, 3 H, H of methyl group of HA), 3.02–3.69 (m, 24.53H, H of HA and C-3, C-5, C-6, C-2, C-4 and methyl group of permethyl-*β*-CD), 4.32–4.39 (m, 2H, H of HA), 5.15–5.32 (m, 0.95H, H of C-1 of permethyl-*β*-CD).

### Synthesis of porphyrin modified paclitaxel prodrug (PorTaxol)

5-(4-carboxyphenyl)-10,15,20- triphenylporphyrin (82.3 mg, 0.125 mmol) was dissolved in chloroform (20 mL), and paclitaxel (106.8 mg, 0.125 mmol), *N,N*′-dicyclohexyl-carbodiimide (DCC) (51.6 mg, 0.25 mmol), and 4-dimethylaminopyridine (DMAP) (10.2 mg, 0.083 mmol) were dissolved in chloroform (10 mL), and then the above mentioned two solutions were mixed together and the mixture was stirred in absence of light at room temperature under nitrogen atmosphere for 24 h. The solution was filtered, and the filtrate was dried under the reduced pressure to remove the solvent. Then the residue was dissolved in chloroform (50 mL) and washed with water (3 × 50 mL), and the organic phase was dried over Na_2_SO_4_. The solvent was removed under the reduced pressure, and the crude product was purified by column chromatography (silica gel, dichloromethane/ethyl acetate from 10/1 to 5/1 as eluent) to give PorTaxol as a a purple solid (84 mg, 45% yield). ^1^H NMR (400 MHz, CDCl_3_, TMS): *δ* = −2.79 (brs, 2H), 1.18 (s, 3H), 1.30 (s, 3H), 1.72 (s, 3H), 1.73–1.74 (m, 1H_*α*_), 2.11 (s, 3H), 2.26 (s, 3H), 2.41–2.47 (m, 2H), 2.54–2.55 (m, 1H_*β*_), 2.57 (s, 3H), 3.89–3.91 (d, *J* = 7.0 Hz, 1H), 4.22–4.25 (d, *J* = 8.4 Hz, 1H_*α*_), 4.35–4.38 (d, *J* = 8.4 Hz, 1H_*β*_), 4.50–4.53 (m, 1H), 5.03–5.05 (m, 1H), 5.72–5.74 (m, 1H), 5.88–5.89 (d, *J* = 3.8 Hz, 1H), 6.17–6.20 (dd, *J* = 9.0, 3.7 Hz, 1H), 6.37 (s, 1H), 6.37–6.43 (t, *J* = 9.6 Hz, 1H), 7.19 (d, *J* = 9.0 Hz, 1H), 7.36–7.70 (m, 15H), 7.74–7.85 (m, 9H), 8.18–8.22 (m, 6H), 8.32–8.34 (d, *J* = 8.2 Hz, 2H), 8.38–8.40 (d, *J* = 8.2 Hz, 2H), 8.76–8.77 (m, 2H), 8.86–8.88 (m, 6H); ^13^C NMR (100 MHz, CDCl_3_, TMS): *δ* = 9.7, 15.1, 20.9, 22.7, 25.0, 25.6, 26.9, 29.4, 29.7, 32.0, 34.0, 43.3, 49.2, 58.6, 72.1, 72.2, 75.2, 75.7, 79.3, 81.1, 84.5, 120.5, 126.8, 127.2, 127.9, 128.2, 128.8, 128.8, 129.3, 130.3, 132.1, 133.8, 134.6, 134.9, 142.0, 143.0, 166.0, 167.1, 168.4, 169.9, 171.3, 203.9 ppm; ESI-MS: *m*/*z*: 1494.5612 [M+H]^+^; elemental analysis calcd (%) for C_92_H_79_N_5_O_15_·8H_2_O: C 67.43, H 5.84, N 4.27; found: C 67.40, H 5.82, N 4.77.

### Preparation and drug loading ratio/drug encapsulation ratio measurements of HApCD-PorTaxol nanoparticles (HATXP)

Porphyrin modified paclitaxel (1.72 mg, 1.15 *μ*mol) in DMSO (500 *μ*L) was added to a solution of HApCD (5 mg, 0.018 *μ*mol, containing 1.14 *μ*mol permethyl-*β*-CD) in deionized water/PBS (25 mL), and then the mixture was ultrasonicated for 5 min. The resulting HATXP solution was dialyzed against excess amount of deionized water for 2 h to remove any unbounded PorTaxol, and the resulting solution was stored at 4 °C. After that the above mentioned solution was extracted by chloroform (3 × 30 mL) until the aqueous phase turned to colorless, then the organic phase was combined and was dried over Na_2_SO_4_, and then the chloroform was removed under reduced pressure. The residue was dissolved in 10 mL DMSO, and the concentration of loaded PorTaxol was measured by UV/Vis spectroscopy and the photometric standard curve of PorTaxol at *λ* = 515 nm.

### Enzyme-triggered drug release experiments

Porphyrin modified paclitaxel prodrug (1.72 mg, 1.15 *μ*mol) in DMSO (500 *μ*L) was added to a solution of HApCD (5 mg, 0.018 *μ*mol, containing 1.14 *μ*mol permethyl-*β*-CD) in deionized water (25 mL), and then the mixture was ultrasonicated for 5 min. After that, hyaluronidase was added (the final concentration of hyaluronidase was 0.5 IU mL^−1^), and the solution was stirred for 2 h at 37 °C. The obtained solution was subjected to the examinations by DLS, TEM and Tyndall effect.

### Cytotoxicity experiments

SKOV-3 human ovarian cancer cells were cultured in the McCoy’s 5A medium, and NIH3T3 mouse embryo fibroblasts were cultured in the Dulbecco-modified Eagle’s medium (DMEM), which were both supplemented with 10% fetal calf serum (FCS) in 96-well plates (3 × 10^4^ cells mL^−1^, 100 *μ*L medium per well) for 24 h. The cells were incubated with Taxol, PorTaxol prodrug, HATXP, HATXP with excess HA, and HApCD ([Taxol] = [PorTaxol] = 20 *μ*M, [HApCD] = 0.34 *μ*M, [HA] = 14.8 *μ*M). After incubation for 24 and 48 h, the relative cellular viability was measured by the MTT assays. All the data are presented as the mean ± standard deviation.

### Fluorescent confocal imaging

SKOV-3 human ovarian cancer cells and NIH3T3 mouse embryo fibroblasts were cultured in the McCoy’s 5A medium, and in the DMEM, respectively, which were both supplemented with 10% fetal calf serum (FCS) in 6-well plates (5 × 10^4^ cells mL^−1^, 2 mL medium per well) for 24 h. Then the cells were incubated with HATXP ([PorTaxol] = 20 *μ*M, [HApCD] = 0.34 *μ*M), After incubation for 6 h, the culture medium was removed and the cells were washed with fresh PBS for 3 times, and then subjected to the observation by a fluorescence confocal microscope.

Statistical analysis of the data was carried out by using the Student’s t-test. Differences were considered statistically significant if the P value was < 0.05.

## Additional Information

**How to cite this article**: Yang, Y. *et al.* Polysaccharide-based Noncovalent Assembly for Targeted Delivery of Taxol. *Sci. Rep.* **6**, 19212; doi: 10.1038/srep19212 (2016).

## Supplementary Material

Supplementary Information

## Figures and Tables

**Figure 1:**
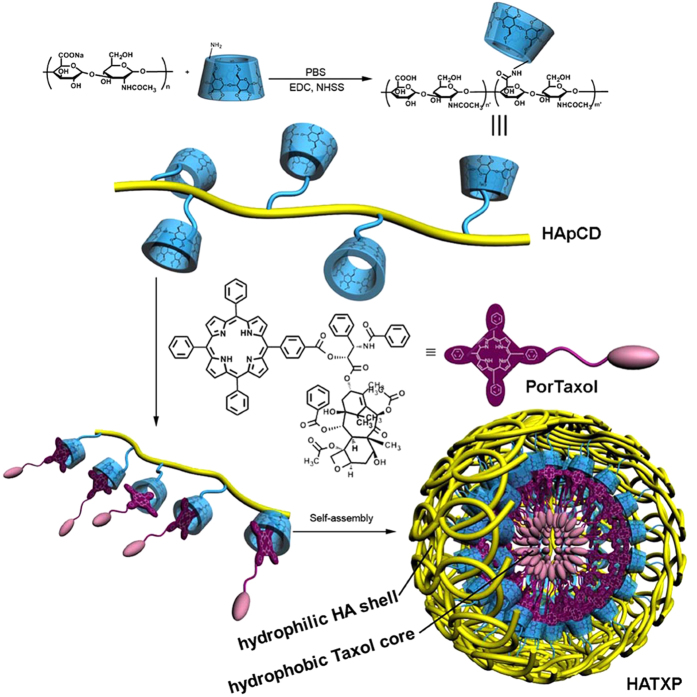
The synthetic routes of HApCD, PorTaxol, and HATXP nanoparticle. HApCDs were synthesized by side-chain modification using amide condensation reaction.

**Table 1:** Characterization of HA and HApCD.

Sample	Feed ratio^a^	*M*_w_’^b^	*M*_*w*_^c^	*M*_n_^d^	*M*_w_/*M*_n_	DS	R_h_ (nm)^e^
HA	0	1.90 × 10^5^^f^	1.94 × 10^5^	1.68 × 10^5^	1.15	none	15.8
HApCD	1	2.72 × 10^5^	2.83 × 10^5^	2.58 × 10^5^	1.10	7.37	20.6

^a^Molar feed ratio of permethyl-*β*-CD to sugar residues of HA polymer.

^b^Molecular weights estimated from ^1^H NMR spectra.

^c^Weight-averaged molecular weights obtained from GPC.

^d^Number-averaged molecular weights obtained from GPC.

^e^Hydrodynamic radius of HA and HApCD measured by viscosity detector of GPC in PBS.

^f^Molecular weight obtained according to the specification of hyaluronic acid sodium purchased from commercial sources.
