# Supplementary material for: Polysaccharide-based Noncovalent Assembly for Targeted Delivery of Taxol
Source: Sci Rep. 2016 Jan 13;6:19212. doi: 10.1038/srep19212 (PMC4725941; doi:10.1038/srep19212)
Supplement: Supplementary Information [file srep19212-s1.pdf]

Supplementary Information for

Polysaccharide-based Noncovalent Assembly for Targeted Delivery of Taxol

Yang Yang[3,1], Ying-Ming Zhang[1], Yong Chen[1,2], Jia-Tong Chen[4] & Yu Liu[1,2*]

[1]Department of Chemistry, State Key Laboratory of Elemento-Organic Chemistry, Nankai University, Tianjin, 300071, P. R. China, E-mail: yuliu@nankai.edu.cn

[2]Collaborative Innovation Center of Chemical Science and Engineering (Tianjin), Nankai University, Tianjin, 300071, P. R. China

[3]School of Chemical Engineering and Technology, Hebei University of Technology, Tianjin, 300130, P. R. China

[4]Department of Biochemistry and Molecular Biology, College of Life Sciences, Nankai University, Tianjin, 300071, P. R. China

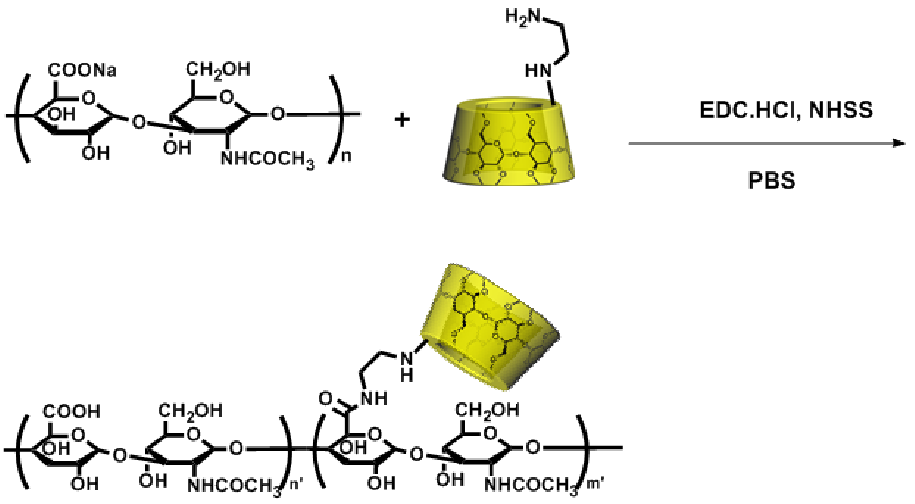

Supplementary Figure S1. The synthetic route of HApCD.

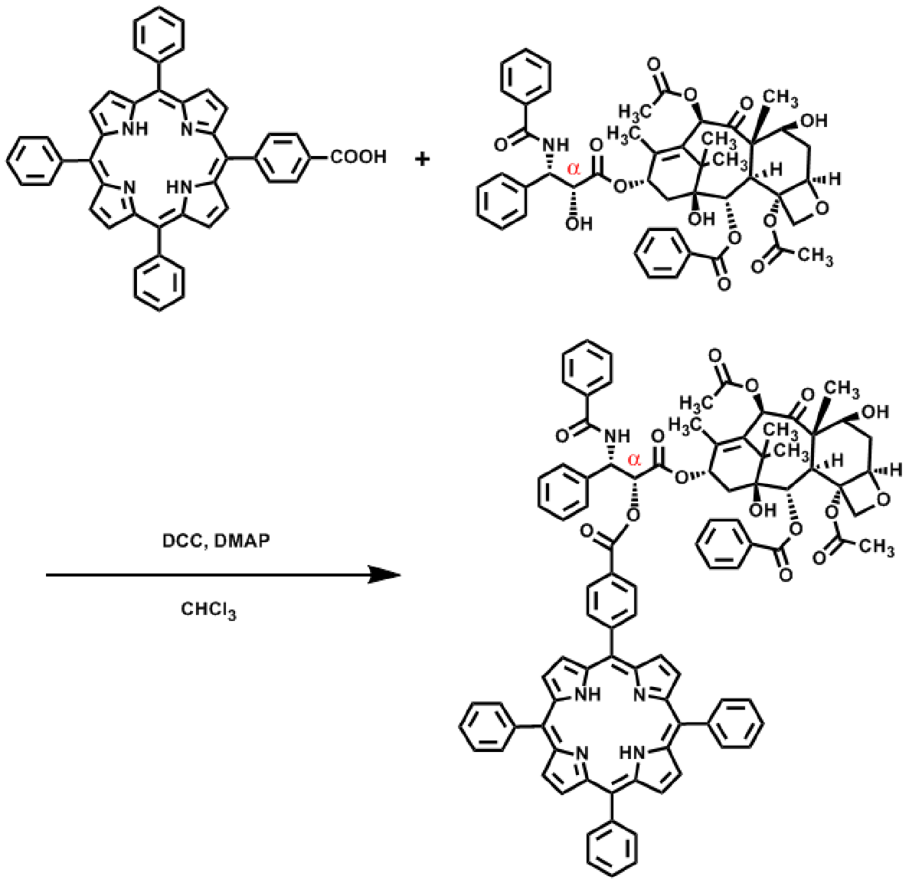

Supplementary Figure S2. The synthetic route of PorTaxol.

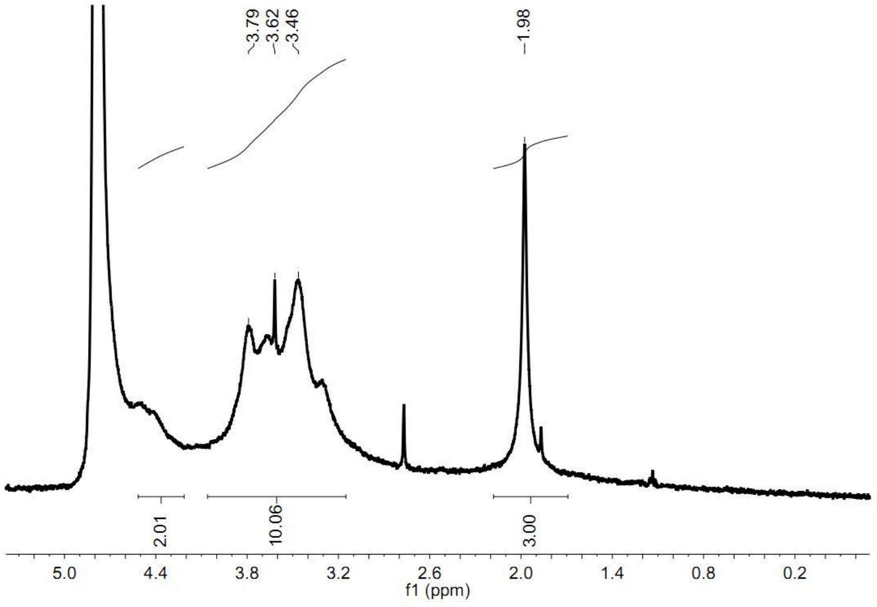

Supplementary Figure S3. ^{1}H NMR (400 MHz) spectrum of HA (M_w = 190,000) in D_2O at 25 ℃.

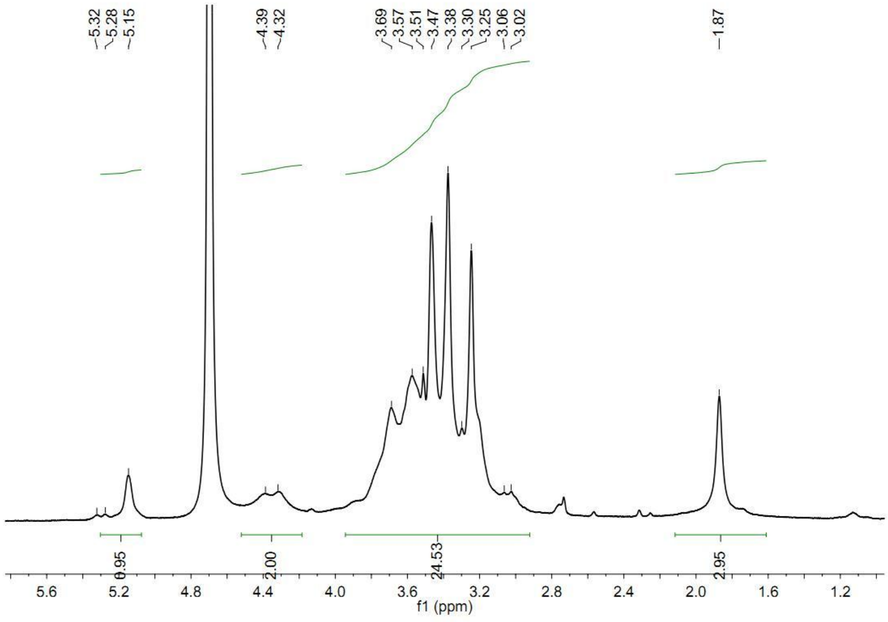

Supplementary Figure S4. ^{1}H NMR (400 MHz) spectrum of HApCD in D_2O at 25 ℃.

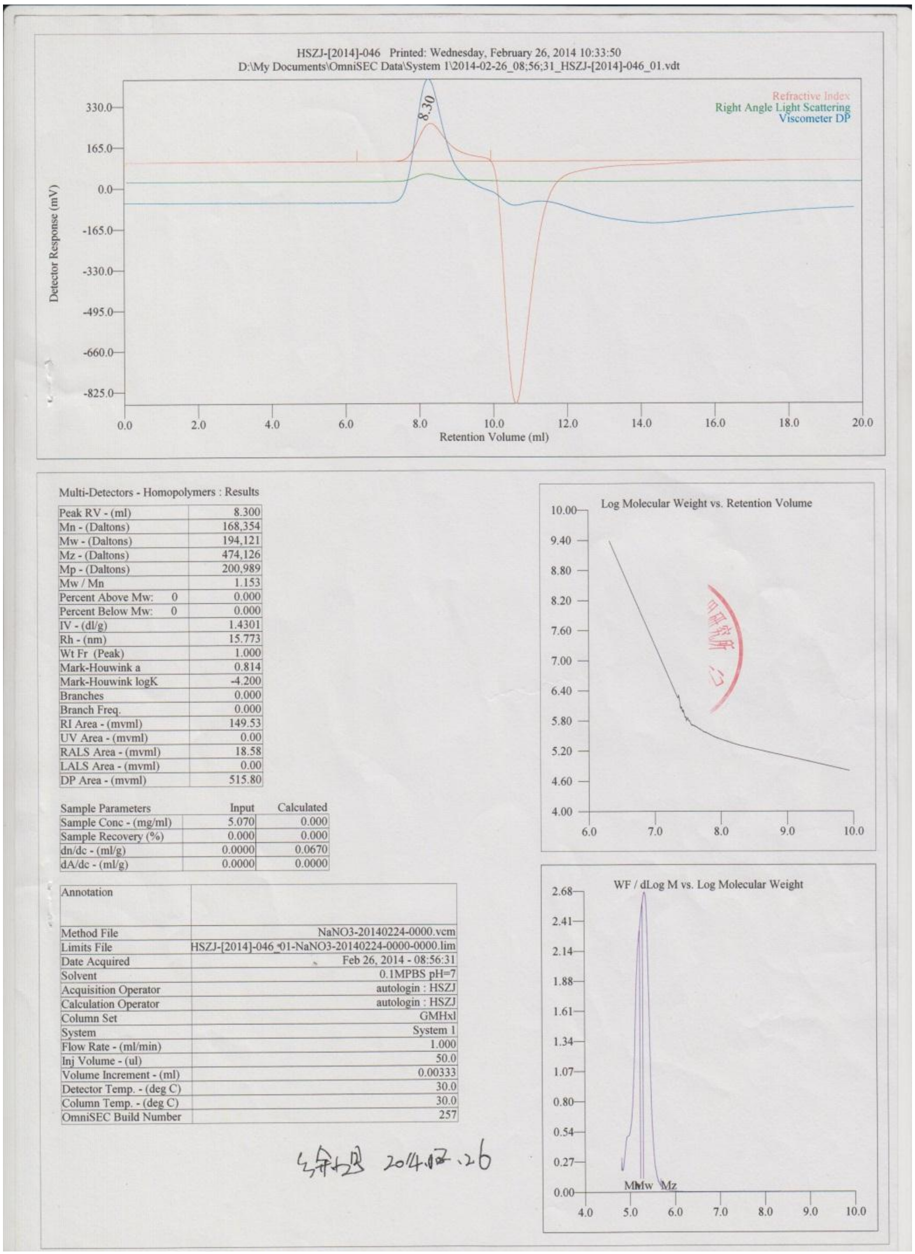

Supplementary Figure S5. GPC results obtained for HA in PBS at the concentration of 5 mg/mL.

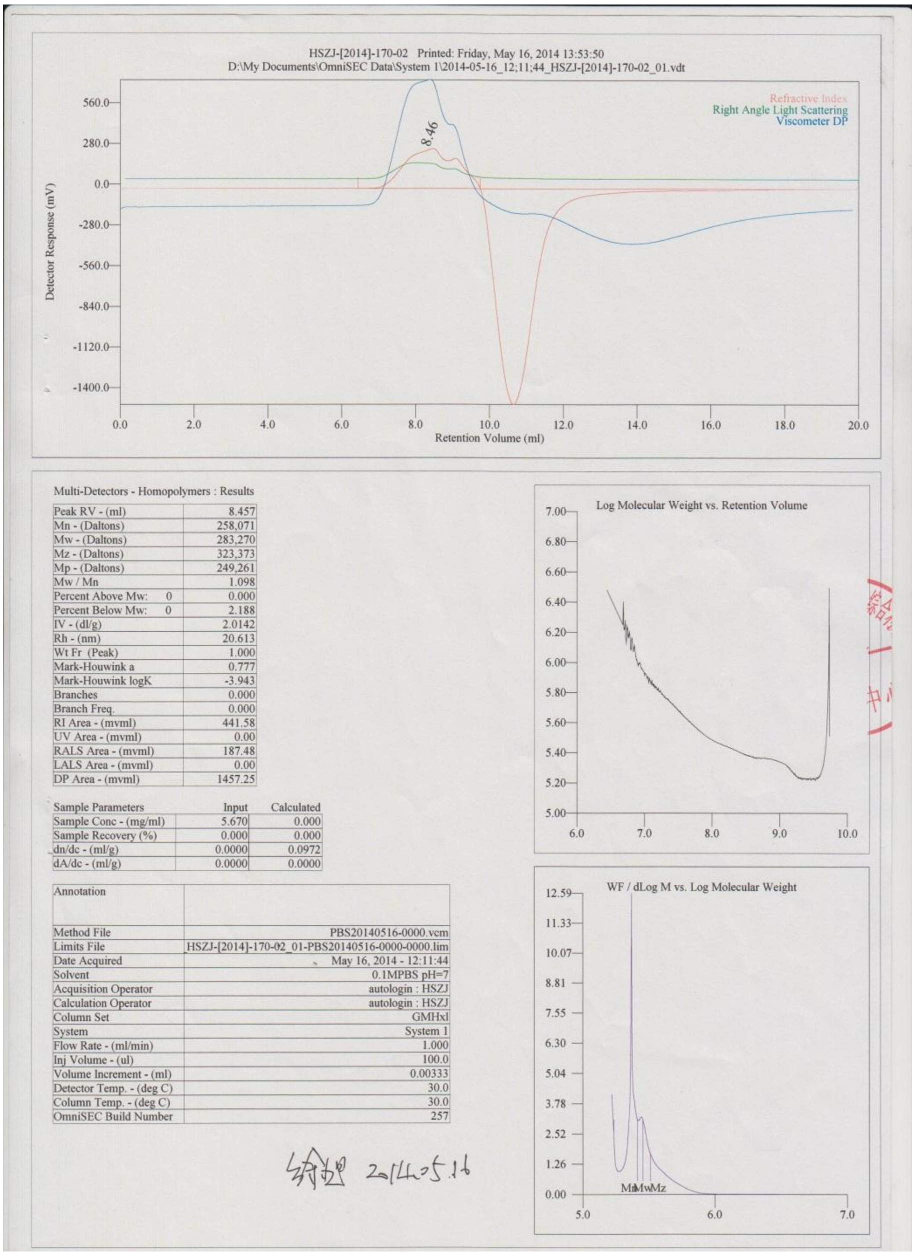

Multi-Detectors - Homopolymers : Results

Peak RV - (ml)	8.457
Mn - (Daltons)	258,071
Mw - (Daltons)	283,270
Mz - (Daltons)	323,373
Mp - (Daltons)	249,261
Mw / Mn	1.098
Percent Above Mw: 0	0.000
Percent Below Mw: 0	2.188
IV - (dl/g)	2.0142
Rh - (nm)	20.613
Wt Fr (Peak)	1.000
Mark-Houwink a	0.777
Mark-Houwink logK	-3.943
Branches	0.000
Branch Freq.	0.000
RI Area - (mvml)	441.58
UV Area - (mvml)	0.00
RALS Area - (mvml)	187.48
LALS Area - (mvml)	0.00
DP Area - (mvml)	1457.25

Sample Parameters	Input	Calculated
Sample Conc - (mg/ml)	5.670	0.000
Sample Recovery (%)	0.000	0.000
dn/dc - (ml/g)	0.0000	0.0972
dA/dc - (ml/g)	0.0000	0.0000

Annotation	
Method File	PBS20140516-0000.vcm
Limits File	HSZJ-[2014]-170-02_01-PBS20140516-0000-0000.lim
Date Acquired	May 16, 2014 - 12:11:44
Solvent	0.1MPBS pH=7
Acquisition Operator	autologin : HSZJ
Calculation Operator	autologin : HSZJ
Column Set	GMHxl
System	System 1
Flow Rate - (ml/min)	1.000
Inj Volume - (ul)	100.0
Volume Increment - (ml)	0.00333
Detector Temp. - (deg C)	30.0
Column Temp. - (deg C)	30.0
OmniSEC Build Number	257

2014.05.16

Supplementary Figure S6. GPC results obtained for HApCD in PBS at the concentration of 5 mg/mL.

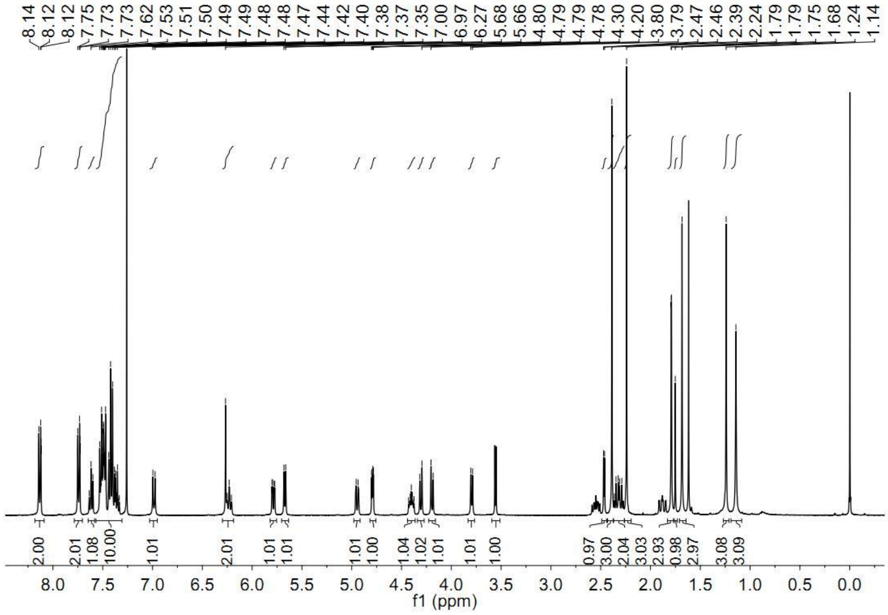

Supplementary Figure S7. ^{1}H NMR (400 MHz) spectrum of Taxol in $CDCl_3$ at 25 ℃.

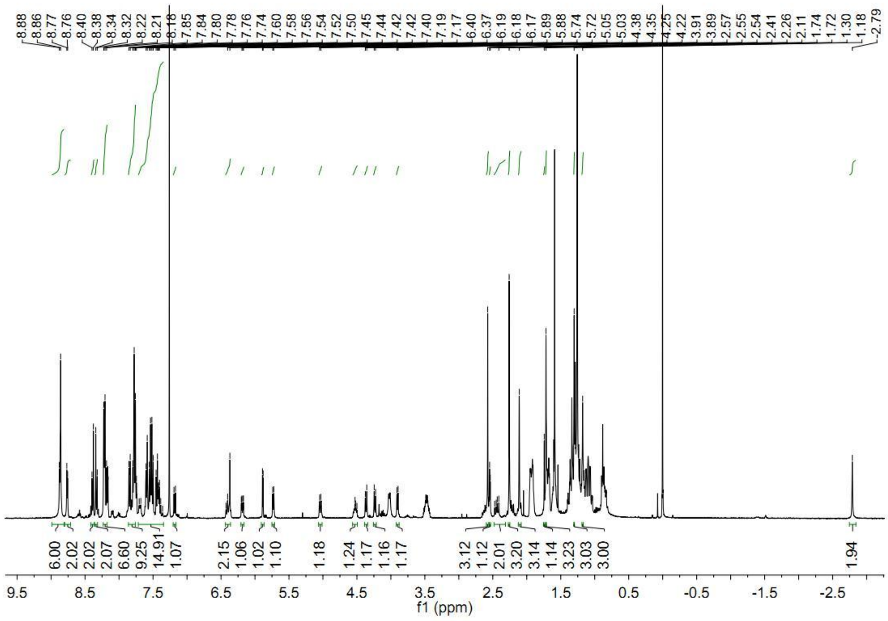

Supplementary Figure S8. ^{1}H NMR (400 MHz) spectrum of PorTaxol prodrug in $CDCl_3$ at 25 ℃.

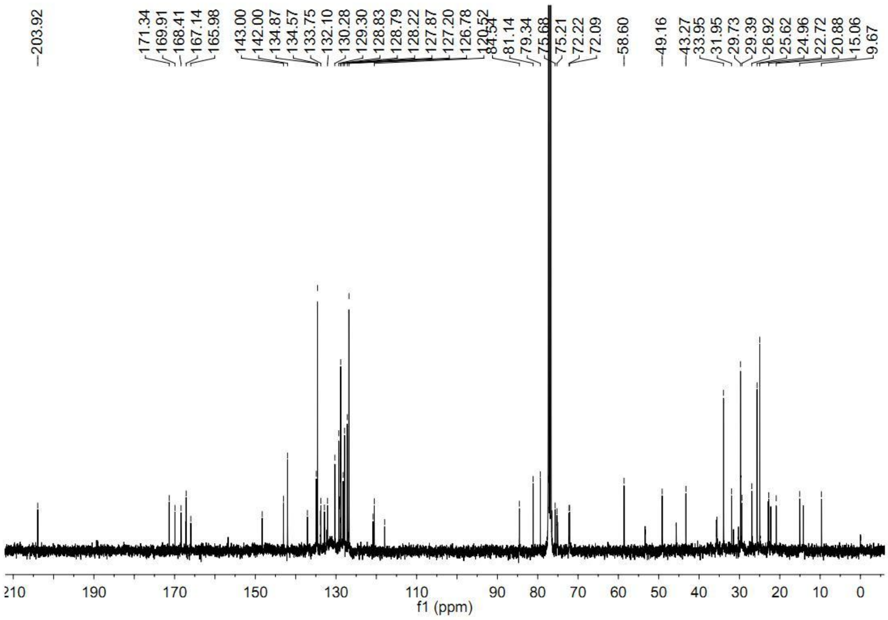

Supplementary Figure S9. ^{13}C NMR (100 MHz) spectrum of PorTaxol prodrug in $CDCl_3$ at 25 ℃.

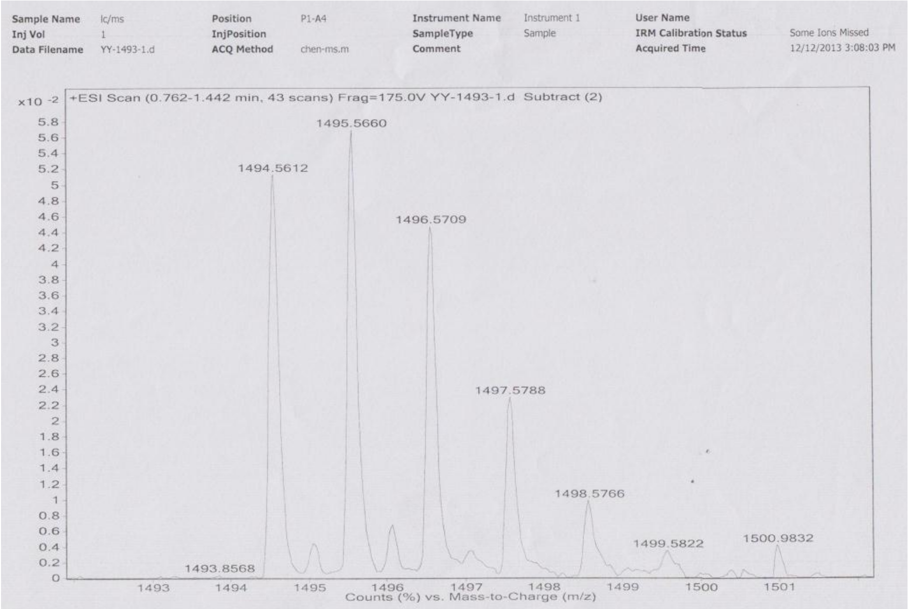

Supplementary Figure S10. ESI mass spectrum of PorTaxol prodrug.

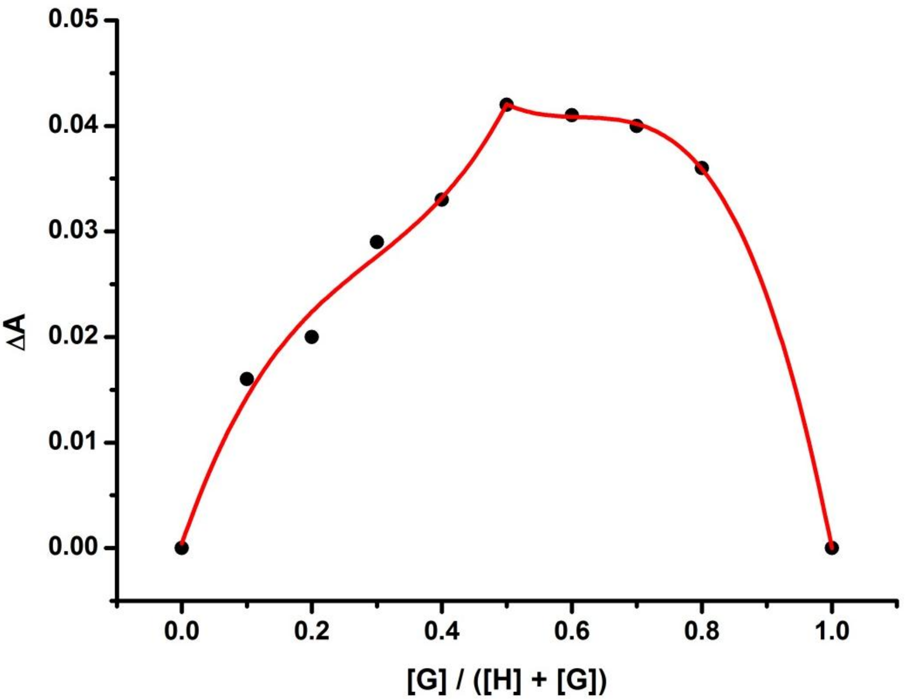

Supplementary Figure S11. The Job's plot of PorTaxol prodrug (G) and permethyl-β-CD (H) in PBS containing 5% DMSO ([PorTaxol] + [permethyl-β-CD] = 12 μM) at 25 ℃.

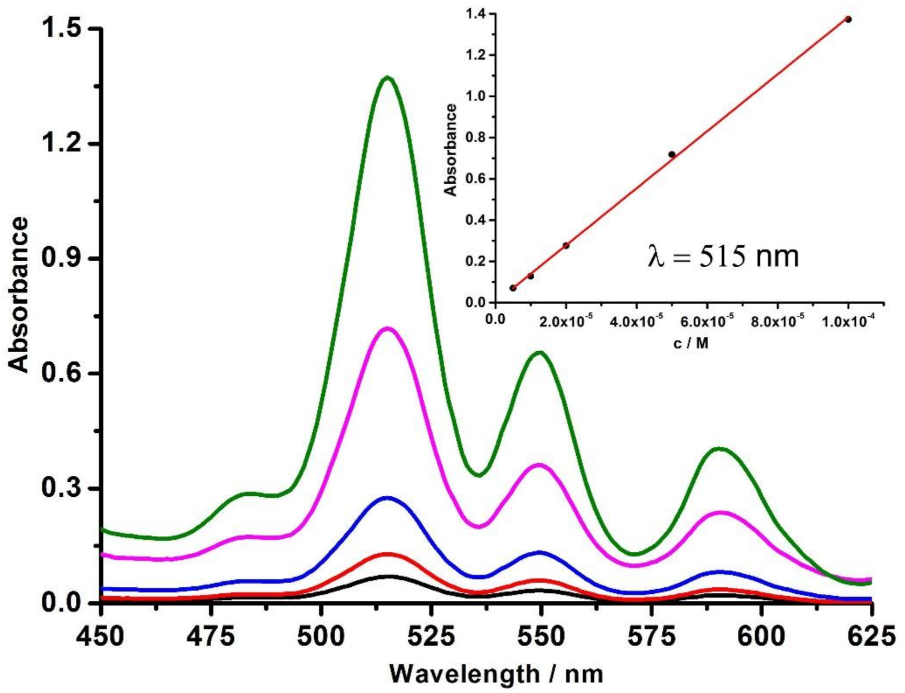

Supplementary Figure S12. UV/Vis spectra of PorTaxol at 5, 10, 20, 50, 100 μM in DMSO. Inset: standard curve of PorTaxol with absorption at λ = 515 nm vs. concentration.

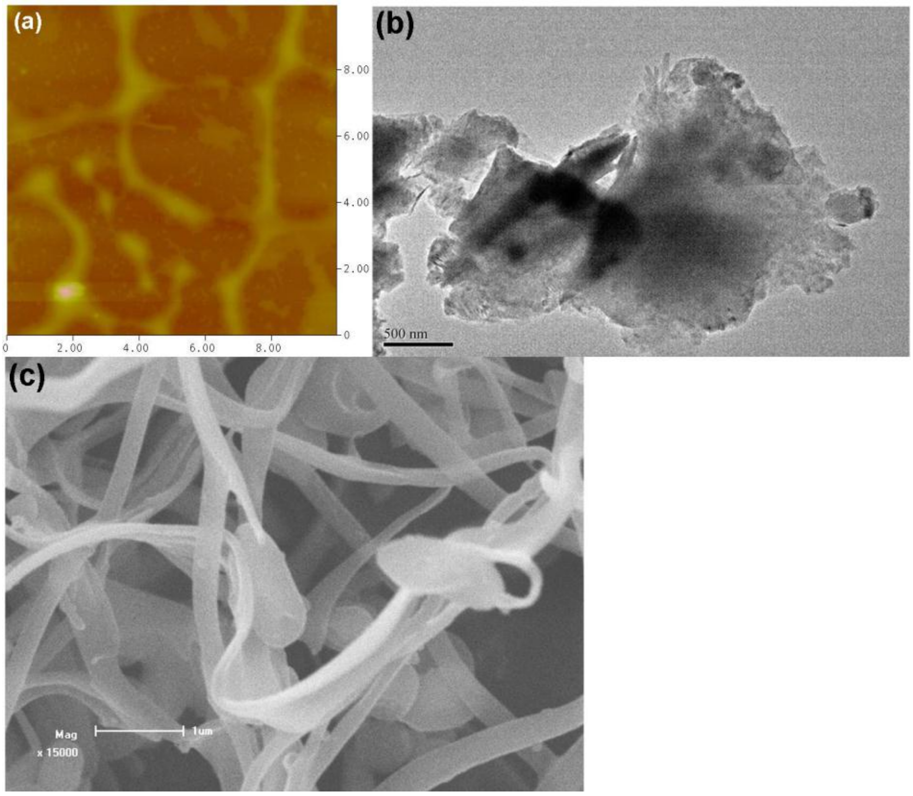

Supplementary Figure S13. The (a) AFM, (b) HR-TEM, (c) SEM images of HApCD.

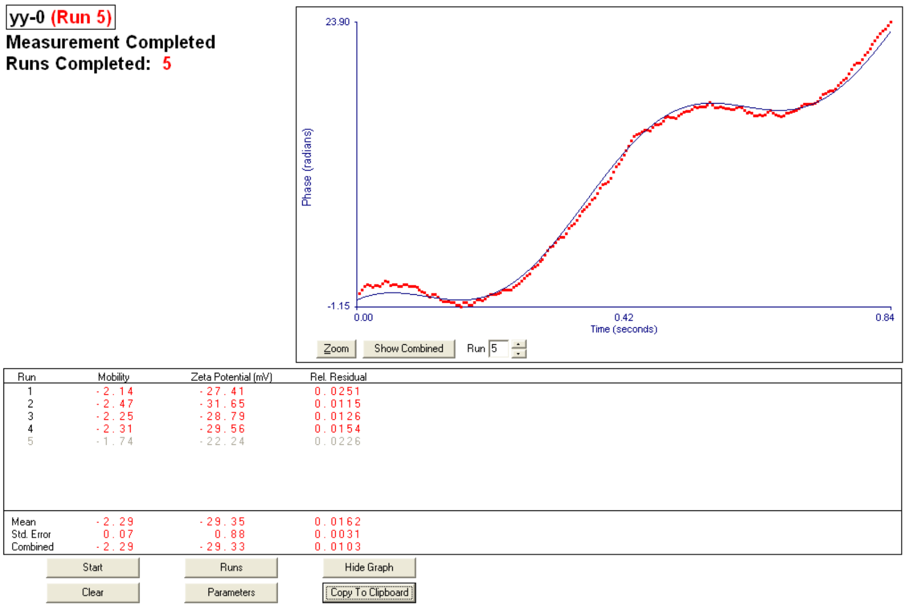

Run	Mobility	Zeta Potential (mV)	Rel. Residual
1	-2.14	-27.41	0.0251
2	-2.47	-31.65	0.0115
3	-2.25	-28.79	0.0126
4	-2.31	-29.56	0.0154
5	-1.74	-22.24	0.0226
Mean	-2.29	-29.35	0.0162
Std. Error	0.07	0.88	0.0031
Combined	-2.29	-29.33	0.0103

Supplementary Figure S14. The zeta potential of HATXP as -29.35 mV.

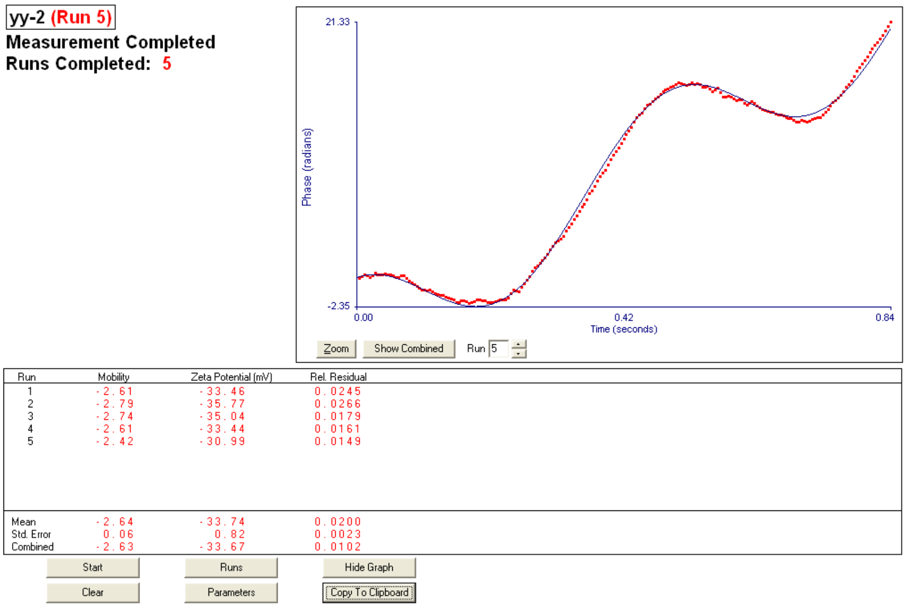

Run	Mobility	Zeta Potential (mV)	Rel. Residual
1	-2.61	-33.46	0.0245
2	-2.79	-35.77	0.0266
3	-2.74	-35.04	0.0179
4	-2.61	-33.44	0.0161
5	-2.42	-30.99	0.0149
Mean	-2.64	-33.74	0.0200
Std. Error	0.06	0.82	0.0023
Combined	-2.63	-33.67	0.0102

Supplementary Figure S15. The zeta potential of HApCD as -33.74 mV.

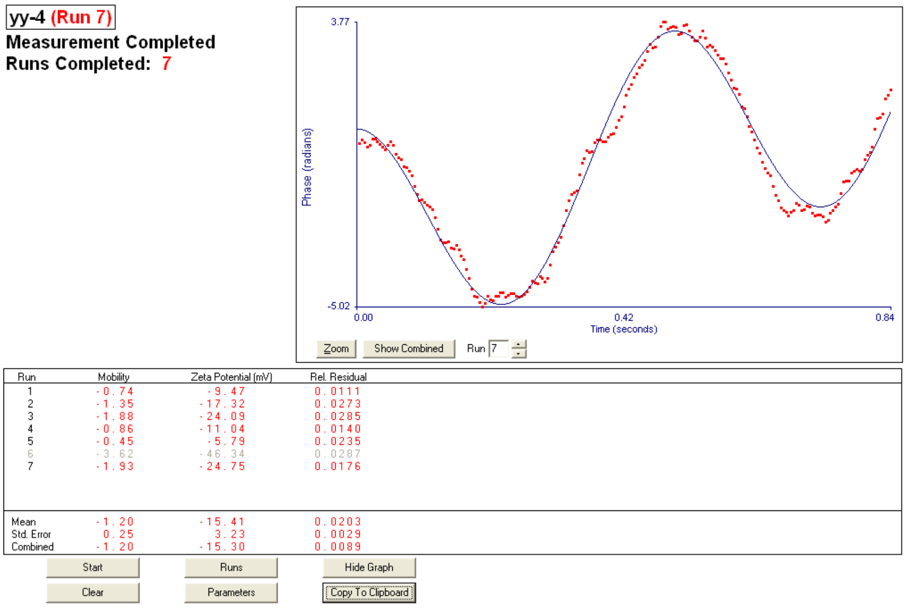

Run	Mobility	Zeta Potential (mV)	Rel. Residual
1	-0.74	-9.47	0.0111
2	-1.35	-17.32	0.0273
3	-1.88	-24.09	0.0285
4	-0.86	-11.04	0.0140
5	-0.45	-5.79	0.0235
6	-3.62	-46.34	0.0287
7	-1.93	-24.75	0.0176
Mean	-1.20	-15.41	0.0203
Std. Error	0.25	3.23	0.0029
Combined	-1.20	-15.30	0.0089

Supplementary Figure S16. The zeta potential of HA as -15.41 mV.

(right).

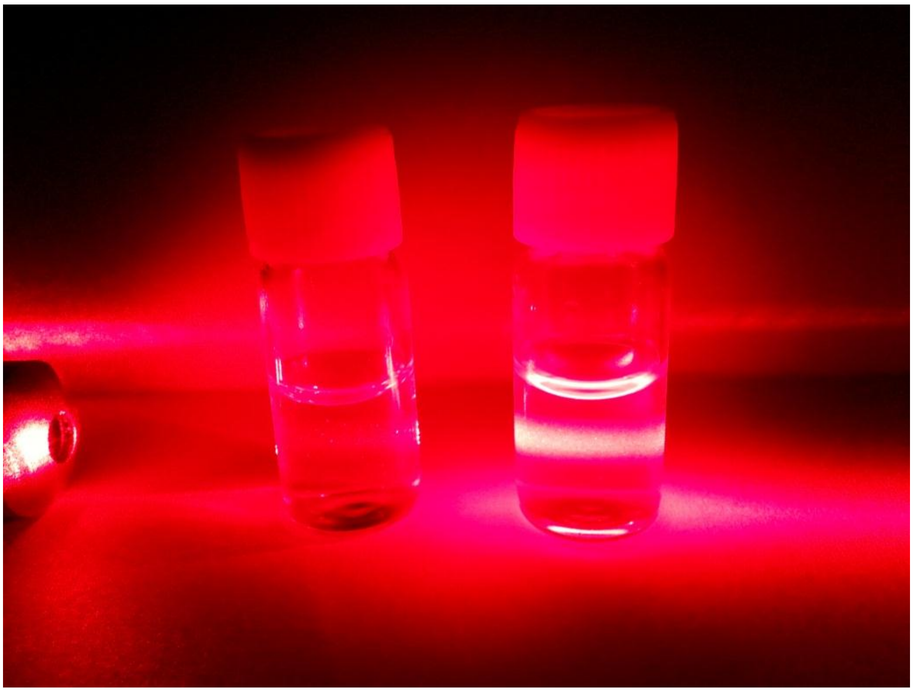

Supplementary Figure S17. The Tyndall effects of HApCD (left) and HATXP (right).

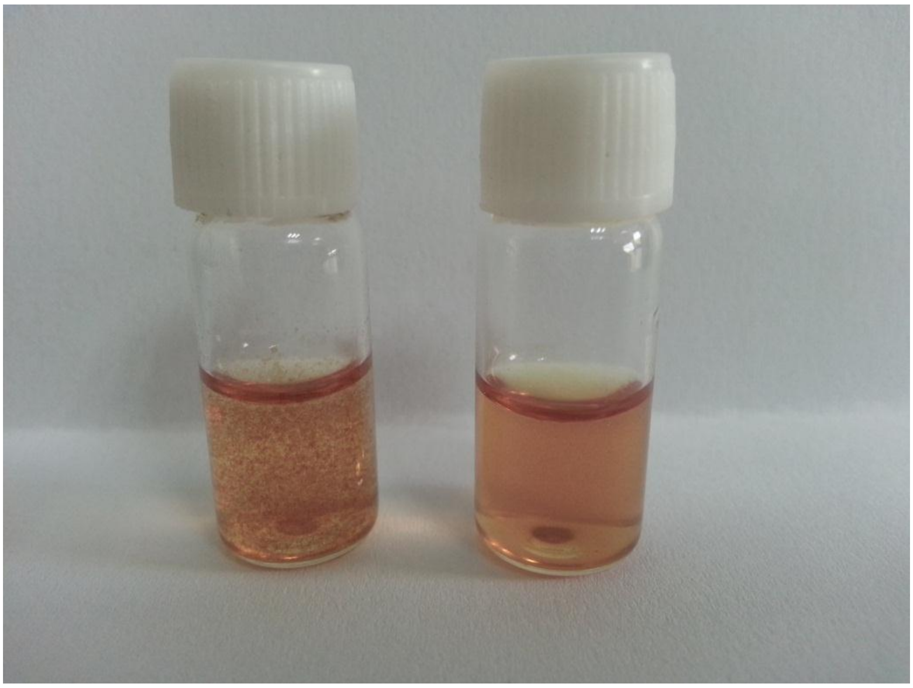

Supplementary Figure S18. The PBS solution of PorTaxol prodrug (left) and HATXP (right).

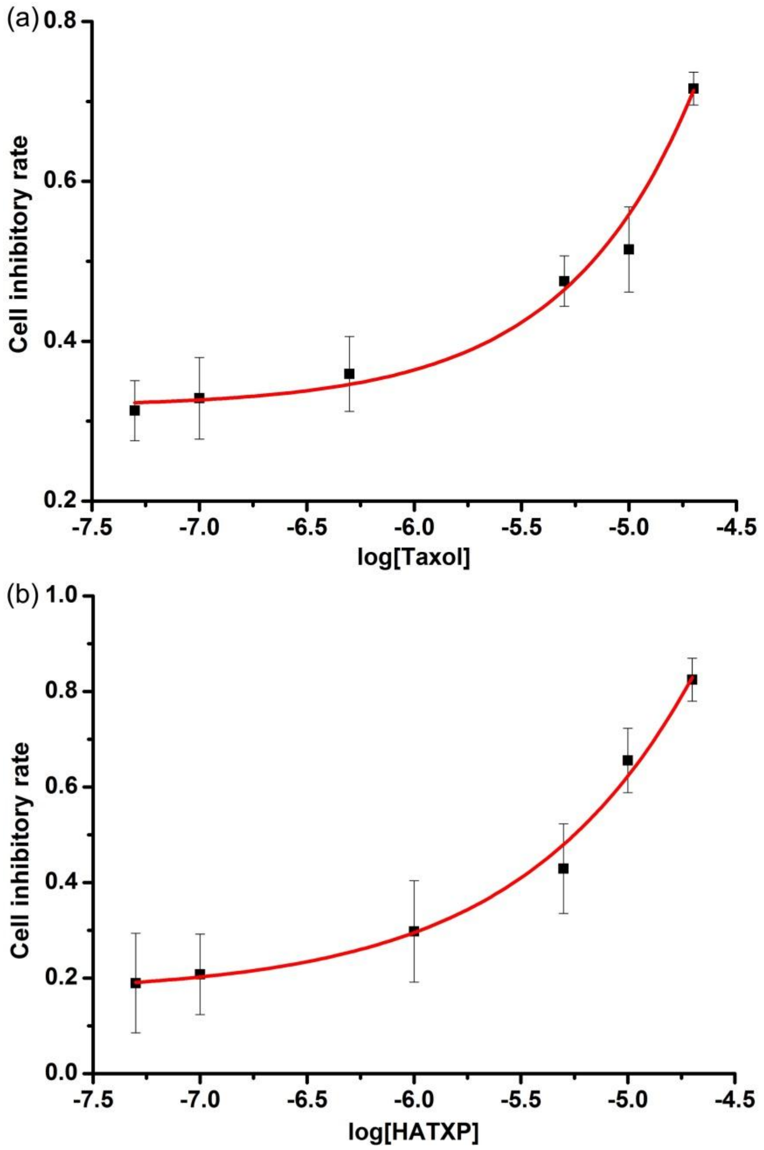

Supplementary Figure S19. The curves of SKOV-3 cell inhibitory rate at different concentrations of (a) Taxol and (b) HATXP.

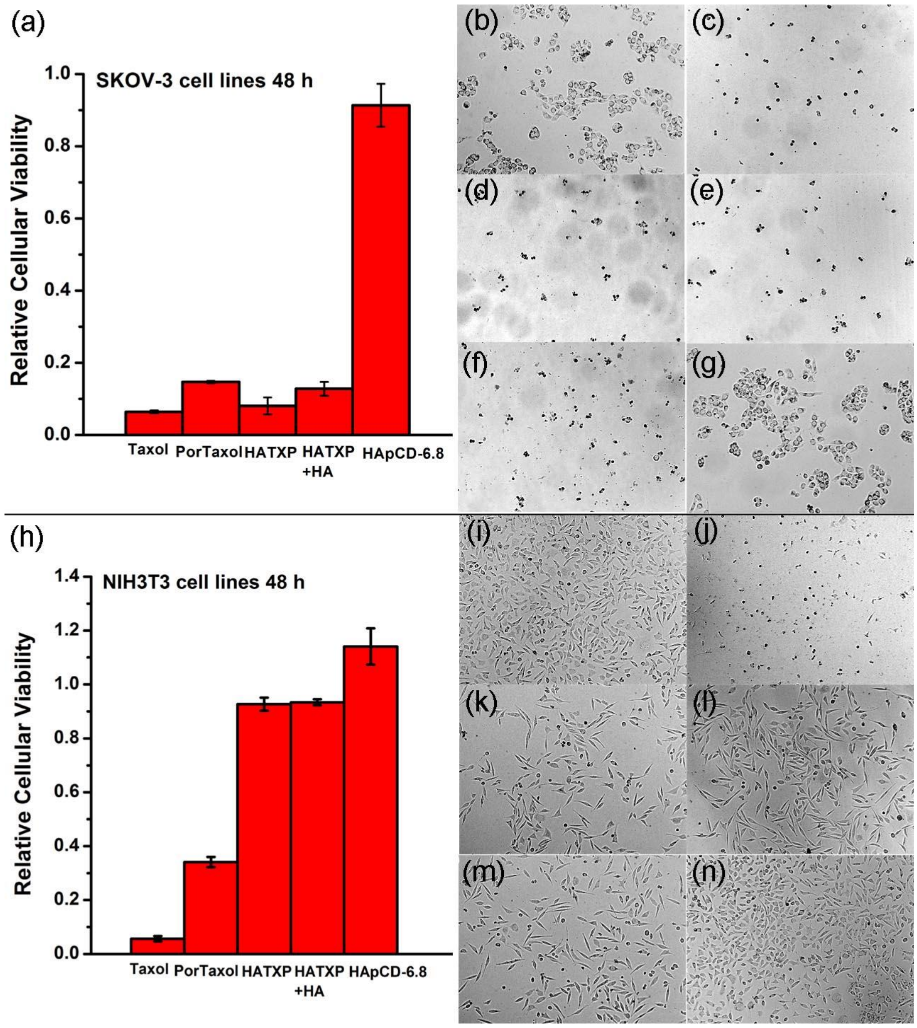

Supplementary Figure S20. Cytotoxicity experiment results of (a) SKOV-3 cells and (h) NIH3T3 cells in 48 h, SKOV-3 cell images of (b) blank control; (c) Taxol; (d) PorTaxol prodrug; (e) HATXP; (f) HATXP+HA; (g) HApCD, and NIH3T3 cell images of (i) blank control; (j) Taxol; (k) PorTaxol prodrug; (l) HATXP; (m) HATXP+HA; (n) HApCD.
